# The overriding role of surgery and tumor grade for long‐term survival in patients with gastroenteropancreatic neuroendocrine neoplasms: A population‐based cohort study

**DOI:** 10.1002/cnr2.1462

**Published:** 2021-06-08

**Authors:** Jon Arne Søreide, Jan Terje Kvaløy, Dordi Lea, Oddvar M. Sandvik, Mohammed Al‐Saiddi, Torjan M. Haslerud, Herish Garresori, Lars N. Karlsen, Einar Gudlaugsson, Kjetil Søreide

**Affiliations:** ^1^ Department of Gastrointestinal Surgery Stavanger University Hospital Stavanger Norway; ^2^ Department of Clinical Medicine University of Bergen Bergen Norway; ^3^ Department of Research Stavanger University Hospital Stavanger Norway; ^4^ Department of Mathematics and Physics University of Stavanger Stavanger Norway; ^5^ Department of Pathology Stavanger University Hospital Stavanger Norway; ^6^ Department of Radiology and Nuclear Medicine Stavanger University Hospital Stavanger Norway; ^7^ Department of Oncology Stavanger University Hospital Stavanger Norway; ^8^ Department of Gastroenterology Stavanger University Hospital Stavanger Norway

**Keywords:** gastrointestinal neuroendocrine tumors, GEP‐NEN, outcomes, surgery, survival, WHO grading

## Abstract

**Background:**

Gastroenteropancreatic neuroendocrine neoplasms (GEP‐NENs) comprise a heterogeneous disease group. Factors that affect long‐term survival remain uncertain. Complete population‐representative cohorts with long‐term follow‐up are scarce.

**Aim:**

To evaluate factors of importance for the long‐term survival.

**Methods and results:**

An Observational population‐based study on consecutive GEP‐NEN patients diagnosed from 2003 to 2013, managed according to national guidelines. Univariable and multivariable survival analyses were performed to evaluate overall survival (OS) and to identify independent prognostic factors. One hundred ninety eligible patients (males, 58.9%) (median age, 60.0 years; range, 10.0–94.2 years) were included. The small bowel, appendix, and pancreas were the most common tumor locations. The World Health Organization (WHO) tumor grade 1–3 distributions varied according to the primary location and disease stage. Primary surgery with curative intent was performed in 66% of the patients. The median OS of the study population was 183 months with 5‐ and 10‐year OS rates of 66% and 57%, respectively. Only age, WHO tumor grade, and primary surgical treatment were independent prognostic factors for OS.

**Conclusion:**

The outcomes of GEP‐NEN patients are related to several factors including age and primary surgical treatment. WHO tumor grading, based on the established criteria, should be routine in clinical practice. This may improve clinical decision‐making and allow the comparison of outcomes among different centers.

## 1 INTRODUCTION

Gastroenteropancreatic neuroendocrine neoplasms (GEP‐NENs) are neuroendocrine tumors characterized by heterogeneous clinical patterns, a relatively indolent growth rate, and the ability to secrete peptide hormones and biogenic amines.^1^, ^2^, ^3^ Historically, GEP‐NENs were thought to be relatively rare, but recent reports from different regions suggest a higher and increasing annual GEP‐NEN incidence.^3^, ^4^, ^5^, ^6^, ^7^ Many patients with well‐differentiated neuroendocrine neoplasms, even those with advanced disease at the time of diagnosis, can survive for several years.^5^, ^8^, ^9^, ^10^ In terms of the *prevalence* in this group of patients, GEP‐NENs are the most common gastrointestinal malignancy after colorectal cancer.^1^ This translates into a considerable number of patients requiring long‐term surveillance and treatment.

An enhanced understanding of the biology of this disease and the development of novel diagnostic approaches (i.e., molecular detection, receptor‐based approaches, and metabolic positron emission tomography [PET]) and treatment options (i.e., biological or targeted treatments and improved surgical approaches) have increased the complexity of the clinical management of neuroendocrine tumors.^3^, ^11^ Some of these efforts have likely contributed to an improved survival rate of subgroups of GEP‐NEN patients.^5^, ^8^

This study aimed to evaluate the long‐term survival in a population‐based cohort of consecutive GEP‐NEN patients treated in routine practice who were classified according to current grading and staging criteria of the given time period.^12^, ^13^

## 2 MATERIALS AND METHODS

This observational study included all GEP‐NEN patients treated at a single hospital that covers a geographically well‐defined area. The manuscript follows the Strengthening the Reporting of Observational Studies in Epidemiology (STROBE) statement.^14^

### 2.1 Study population

The study population comprised unselected consecutive patients with GEP‐NENs diagnosed at the Stavanger University Hospital between 2003 and 2013, as reported previously in greater detail.^4^ This is the only hospital that serves a regional area of approximately 380 000 inhabitants; thus, patients consisted of a population‐representative cohort from the Norwegian southwest coast and a mixed urban‐rural area.

We excluded nine patients with an unknown primary tumor location. Moreover, three patients with esophageal and two patients with bile duct primary tumors were excluded due to small sample sizes. This study does not include mixed neuroendocrine neoplasm (MINEN). Also, Goblet cell carcinoma as a specifically defined entity was not included. Thus, the final study population included 190 consecutive patients.

### 2.2 Clinical workup for primary treatment decision‐making

Routine evaluation of patients encompassed clinical examination, necessary blood tests including tumor marker detection (i.e., chromogranin A [CgA]), and standard oncologic imaging (i.e., multiphase computed tomography [CT], magnetic resonance imaging [MRI], and somatostatin receptor‐scintigraphy [SRS]), as described in available guidelines.^15^, ^16^, ^17^, ^18^ Positron emission tomography (PET) imaging (^68^Ga‐DOTA‐somatostatin analog‐PET/CT) and metabolic PET‐imaging were not routinely performed. Transthoracic echocardiography was used for suspected carcinoid heart disease. Endoscopy, including endoscopic ultrasound (EUS) and video capsule endoscopy, were available if indicated. Diagnostic step sequences and appropriate adjustments were made according to the clinical presentation, for example, in symptomatic patients, adequate diagnostic steps were performed to locate the primary tumor and to evaluate the disease stage. In contrast, if a gastroenteropancreatic neuroendocrine tumor (GEP‐NET) was incidentally discovered during surgery for a tentative diagnosis (i.e., indication for surgery) other than GEP‐NET, a postoperative evaluation was performed.

### 2.3 Classification and staging according to morphology

As previously described,^4^ patients were originally staged according to the 2009 Union for International Cancer Control (UICC) Tumor‐Node‐Metastasis (TNM) classification,^12^ and the primary tumors were graded according to the 2010 World Health Organization (WHO) classification.^13^ In the present study, the most current criteria^19^ (Table 1) were applied by two pathologists (D.L. and E.G.) during the independent re‐grading of the tumors and included the novel distinction between grade 3 neuroendocrine tumors (NET) and grade 3 neuroendocrine carcinomas (NEC).

**TABLE 1:** WHO grading of neuroendocrine tumors (WHO 2019)^a^

	Grade	Mitotic activity, per 2 mm^2*^	Ki‐67%*
NET Grade 1	Low	1	<3
NET Grade 2	Intermediate	2‐20	3‐20
NET Grade 3	High	>20	>20
LCNEC	High^†^	>20	>20
SCNEC	High^†^	>20	>20
MiNEN	Variable	Variable	Variable

LCNEC, Large‐cell neuroendocrine carcinoma; MiNEN, Mixed neuroendocrine–non‐neuroendocrine neoplasm; NEC, Neuroendocrine carcinoma; NET, Neuroendocrine tumor; SCNEC, Small‐cell neuroendocrine carcinoma.

^*^ Mitotic rates are expressed as the number of mitoses/2 mm^2^ as determined by counting in 50 fields of 0.2 mm^2^ (i.e., in a total area of 10 mm^2^); the Ki67 proliferation index value is determined by counting at least 500 cells in the regions of highest labelling (hot spots), which are identified at scanning magnification.

^†^ Poorly differentiated NECs are not formally graded but are considered high‐grade by definition.

^a^ also ENETS^20^ recommends that ≤2 should be replaced by <3 to include decimal numbers between 2 and 3. [Correction added on 25 June 2021, after first online publication: In the original published version, Table 1 was based on WHO 2010 and has now been updated using 2019 criteria.]

### 2.4 Primary surgical treatment and treatments for advanced disease

Surgical resection of the primary tumor with curative intent was performed whenever possible. During the entire study period, we embraced the strategy of removing a primary small intestinal NET, even in cases of confirmed unresectable liver metastasis.^21^, ^22^ Moreover, palliative surgical treatment was considered when it was feasible to relieve symptoms due to tumor obstruction or a tumor mass or to relieve uncontrolled clinical symptoms due to a functional tumor.

Symptom‐ and disease‐oriented systemic treatments were offered to patients diagnosed with advanced disease, and biological therapy with long‐acting somatostatin analogs (e.g., octreotide and lanreotide) and interferon α‐2B (IFN) were given for well‐differentiated tumors. In contrast, patients with poorly differentiated carcinomatous neuroendocrine carcinomas were offered chemotherapy with various cytotoxic agents in line with principles provided elsewhere.^17^, ^18^, ^23^, ^24^ Liver metastasis‐directed therapies (i.e., hepatic arterial embolization (HAE), radiofrequency ablation (RFA), cytoreductive surgical resection), and peptide receptor radionuclide therapy (PRRT) were offered when indicated. Newer drugs that have been routinely available for more than a decade, such as oral tyrosine kinase inhibitors (sunitinib) and everolimus, or novel combinations of cytotoxic agents (platinum or etoposide‐based therapies, combination therapies with folinic acid, 5‐fluorouracil [5‐FU] or irinotecan [FOLFIRI] or FOLFIRI combined with oxaliplatin [i.e., FOLFIRINOX]), were discussed by a multidisciplinary team and administered in line with current scientific recommendations.^24^

### 2.5 Follow‐up

Follow‐up was conducted by clinical specialists (i.e., primarily surgeons, oncologists, and gastroenterologists) with a particular interest in treating GEP‐NENs. Follow‐up visits were scheduled and completed at the hospital outpatient clinics. Some patients with minimal risk of relapse or those who were radically treated (e.g., surgically treated patients with an incidentally diagnosed tiny appendiceal neuroendocrine tumor of low grade [WHO grade 1] with tumor‐free margins) did not complete scheduled follow‐up in agreement with the Nordic guidelines.^17^, ^24^ The follow‐up visits included clinical screening for any (new) symptoms and a physical examination supplemented by cross‐sectional imaging (mostly computed tomography [CT] with intravenous contrast, MRI or SRS), standard blood biochemistry analysis, and measurement of chromogranin A (CgA) as a tumor biomarker, as previously suggested.^23^ Additional examinations or imaging were performed as indicated. The frequency and components of outpatient visits varied according to the grade of the primary tumor, disease stage, treatment intent, and presence of any suspicious signs of metachronous disease progression, in line with recommendations provided by the Norwegian Neuroendocrine Tumor Group (NNTG)^18^ and international recommendations.^17^, ^23^, ^25^

Any new patient diagnosed with a GEP‐NEN or any patient with relapse or disease progression encountered during follow‐up was assessed by a multidisciplinary team (MDT) to enable decision‐making according to current guidelines. Thus, various treatments were administered to patients with confirmed relapse or those with progressive disease who were receiving systemic therapy for advanced disease. The type of treatments, sequences, combinations, and durations were adjusted according to treatment responses or disease progression according to current treatment principles. Patients in need of treatment options not available at the hospital (e.g., peptide receptor radionuclide therapy [PRRT]) at the time were referred to cooperating centers in Sweden or Denmark.

The management of patients was guided by national and European guidelines, and specifically, the most recent Nordic guidelines for the management of GEP‐NENs (2010^17^ or 2014^24^).

Time and cause of death were obtained from hospital records, which are electronically linked to the Governmental Statistics Norway database (www.ssb.no). Complete follow‐up of all patients was achieved.

### 2.6 Assessment of survival

*Overall survival* (OS) was defined as the number of months from the date of diagnosis to the date of death from any cause or the date of the last follow‐up (May 1, 2020) in surviving patients. Confirmed death due to advanced GEP‐NET was considered an endpoint when the *disease‐specific survival* (DSS) was calculated.

*Relative survival* was calculated as the proportion of patients who survived to a given postoperative time divided by the proportion of individuals of the same age, sex, and year of birth in the general population that would survive to that time. Population survival was calculated using Norwegian population lifetime tables from the Human Mortality Database (HMD, http://www.mortality.org/).

### 2.7 Statistics

The statistical calculations were performed using SPSS version 25 for Mac (IBM, Armonk, NY) and R 3.6.3.^26^ The R‐package “relsurv” version 2.2‐3 was used for the relative survival calculation.^27^

In the descriptive analyses, categorical data are reported as numbers and percentages, and continuous data are reported as medians and ranges or interquartile ranges (IQRs). Non‐parametric tests were used for comparisons between subgroups. OS was estimated by the Kaplan‐Meier method, and the log‐rank test evaluated differences between subgroups. Cox proportional hazard analyses were performed to assess independent predictive factors of OS and DSS. Factors with a *p*‐value < .2 in the univariable analysis were included in the multivariable models, and these selections were run with a backward stepwise model. The results of the Cox regression analyses are expressed as hazard ratios (HRs) with 95% confidence intervals (CIs).

All tests were two‐sided, and a *p*‐value < .050 was considered statistically significant.

## 3 RESULTS

Of 204 patients, 190 (93.1%) patients with a median age of 60.0 (range, 10.0–94.2) years who were diagnosed with GEP‐NENs between 2003 and 2013, were eligible for further evaluation. The clinical characteristics of the study population and the descriptions of GEP‐NENs are provided in Table 2. Small bowel tumors were primarily localized in the ileum. Among the 33 patients with pancreatic NEN, four patients had clinically functional insulinomas.

**TABLE 2:** Patients (*n* = 190) and disease characteristics according to primary treatment approach

	Primary surgery with curative intent, *n* (%)	Palliative or debulking surgery, *n* (%)	No primary surgery, *n* (%)	*p*‐value
Total	126 (66.3)	24 (12.6)	40 (21.1)	
Median age (range), years	55.5 (10.0–90.5)	62.1 (48.0–82.2)	71.9 (48.5–94.2)	<.001
Males (112, 58.9%): Females (78, 41.1%) = 1.44:1	1.33:1	1.18:1	2.07:1	.448
Primary tumor location				<.001
Stomach (*n* = 11)	5 (4.0)	1 (4.2)	5 (12.5)	
Duodenum (*n* = 5)	1 (0.8)	1 (4.2)	3 (7.5)	
Small bowel (*n* = 60)	36 (28.6)	15 (62.5)	9 (22.5)	
Appendix (*n* = 48)	47 (37.3)	0	1 (2.5)	
Colon (*n* = 15)	5 (4.0)	4 (16.7)	6 (15.0)	
Rectum (*n* = 18)	15 (11.9)	1 (4.2)	2 (5.0)	
Pancreas (*n* = 33)	17 (42.4)	2 (8.3)	14 (35.0)	
UICC stage				<.001
I	70 (55.6)	1 (4.3)	6 (15.0)	
II	15 (11.9)	1 (4.3)	0	
III	33 (26.2)	0	4 (10.0)	
IV	8 (6.3)	21 (93.4)	30 (75.0)	
WHO grade 1–3				<.001
G1	87 (69.0)	12(50.0)	10 (25.5)	
G2	30 (23.8)	6 (25.0)	8 (20.0)	
G3	9 (7.1)	7 (28.0)	17 (42.5)	
Unknown	0	0	5 (12.5)	
30‐d mortality	3 (2.4)	2 (8.3)	5 (12.5)	.106
90‐d mortality	8 (6.4)	4 (16.7)	9 (22.5)	.031

While males showed a slight predominance of 58.9%, no significant differences were observed between sexes in the distribution of the primary tumor location. However, in patients whose primary tumors were in the appendix, the median age of 30.4 (range, 10.0–84.9) years was significantly lower than the median age of 62.5 (range, 19.5–90.5) years in patients whose primary tumors were in other locations (*p* < .001).

**FIGURE 1:**
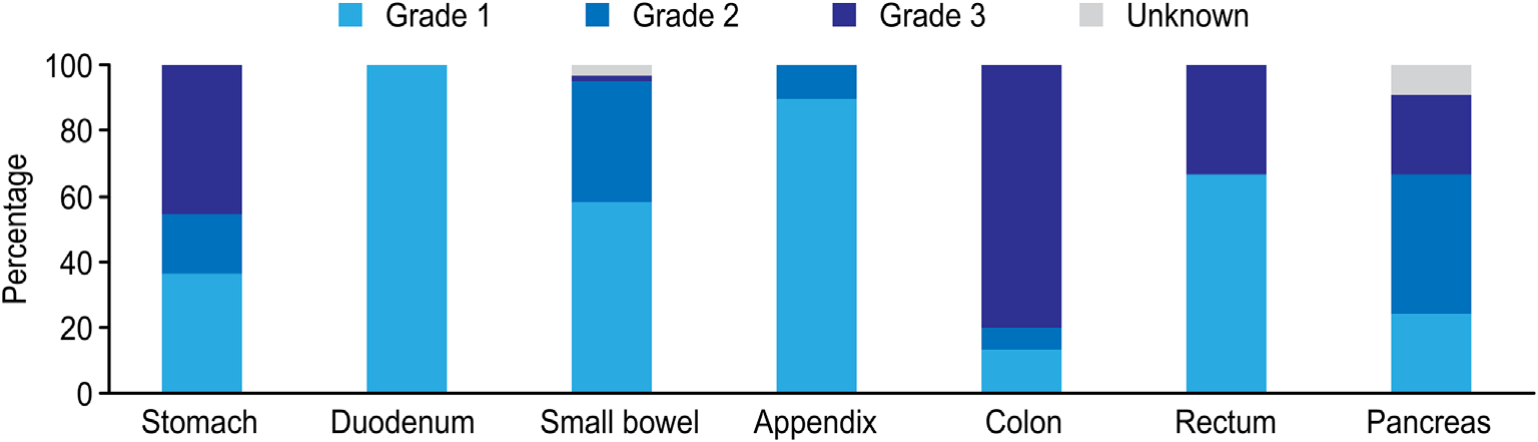
Distribution of WHO tumor grades 1–3 according to primary tumor localization

**FIGURE 2:**
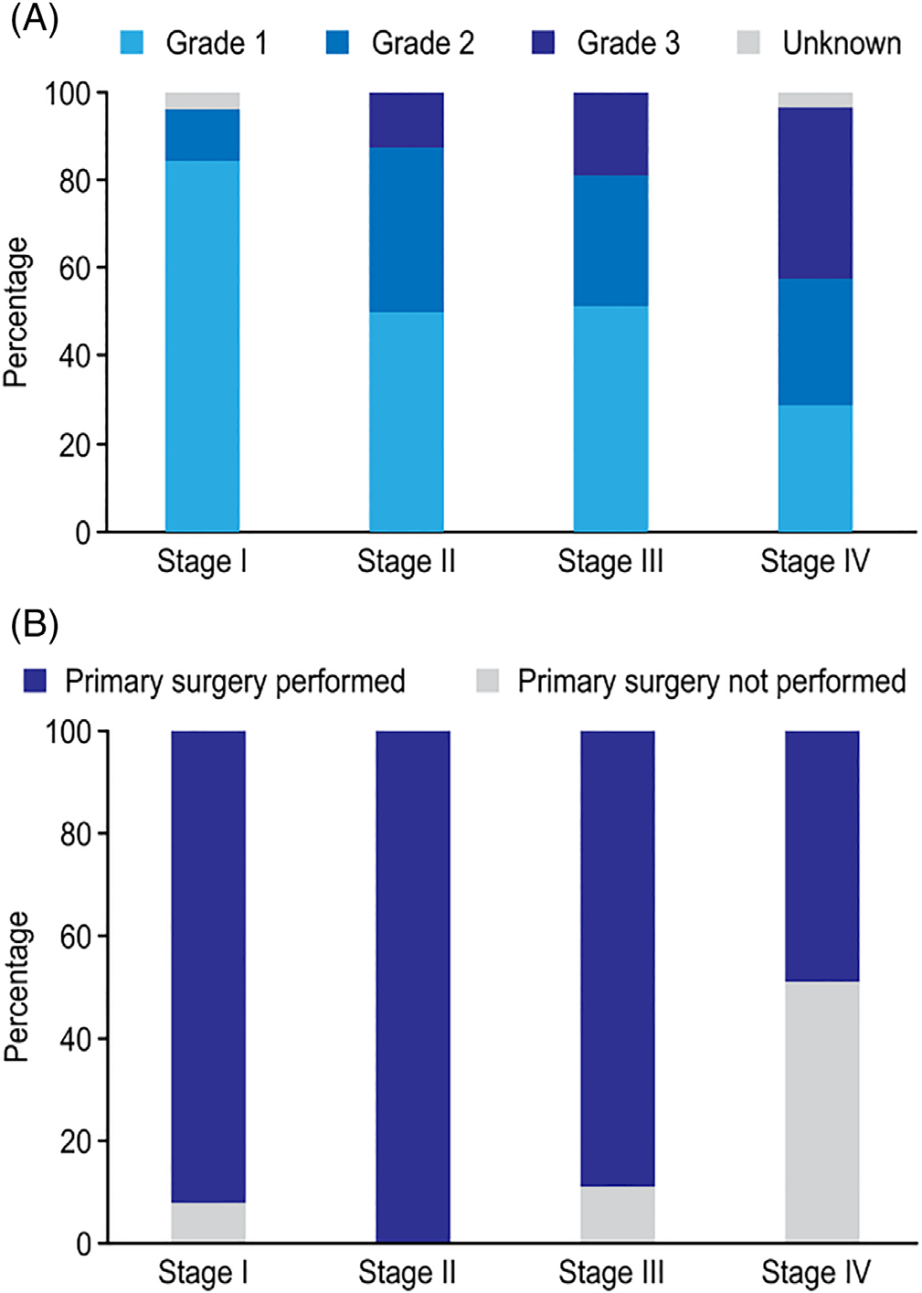
(A) Distribution of WHO tumor grades 1–3 according to disease stage (I–IV). (B) Variations in primary surgical treatment by disease stage (I–IV)

Disease characteristics are displayed as WHO tumor grades 1–3 according to the primary tumor location in Figure 1 and UICC disease stage in Figure 2(A). After the re‐grading of tumors, 179 patients (94.2%) had the same tumor grade. Moreover, nine (4.7%) tumors previously classified as grade 1 became grade 2, and eight (4.2%) tumors previously classified as grade 2 were categorized as grade 1 tumors. All grade 3 tumors remained unchanged.

Grade 2/3 primary tumors were more often found in the colon, pancreas, or stomach. A larger proportion of grade 2/3 tumors was found in patients diagnosed with stage III and IV disease. Of the 42 patients with grade 3 tumors, 7 (16.7%) patients had well‐differentiated NET G3 tumors (i.e., pancreas *n* = 3, colon *n* = 2, small bowel *n* = 1, and unknown primary location *n* = 1), and the remaining 35 patients had neuroendocrine carcinomas (NEC). A small‐cell tumor type (SCNEC) was found in 14 (40%), while the remaining were large‐cell type (LCNEC) neuroendocrine carcinomas.

At the time of diagnosis, resection was performed in 150 patients (78.9%), and of those, 66.3% (126/150) underwent surgery with curative intent (i.e., R0‐resection with tumor‐free margins). Curative surgery was most frequently performed in patients with tumors in the appendix, small bowel, pancreas, and rectum. However, tumors of small bowel origin occurred in a large proportion of patients who underwent palliative or debulking surgery. Due to advanced disease or comorbidities. The remaining 40 patients (21.1%) did not undergo surgery, and small bowel, colon, and pancreas NENs were commonly observed in this group of patients (Table 2). Causes for not having surgery were explained by advanced disease in 57.5% (23/40) of the patients, and patient related factors (i.e., mostly significant comorbidity) were decisive for 13 (32.5%) patients. An unknown cause was encountered in 4 (10%) patients. No significant differences (*p* = .147) were observed between genders with regard to causes for not having primary surgery.

Surgical treatment according to the UICC disease stage is shown in Figure 2(B), and primary surgery was performed significantly more often in patients with stage I–III disease than in patients with stage IV disease (*p* < .001).

The 30‐days mortality varied among treatment groups, with the lowest mortality of 2.4% observed in patients who underwent surgery with curative intent (Table 2). Although the early mortality was higher after palliative surgery and highest when no primary surgery could be performed, these differences did not reach statistical significance.

In contrast, the 90‐days mortality was significantly different among groups (*p* = .031), with threefold increased mortality (16.7%) in patients who underwent palliative surgery. Patients who did not undergo surgery had a fourfold higher mortality rate (22.5%) than patients treated with curative intent (a 90‐day mortality rate of 6.4%) (Table 2).

As a part of primary treatment, 33 (17.4%) patients received biological treatments (i.e., somatostatin analogs), and 21 (11.1%) patients received systemic chemotherapy. Moreover, several additional therapies (i.e., HAE, targeted therapy, or PRRT‐treatment) were administered in some patients. Single or sequentially administered treatments, sometimes in combination, were used as indicated and were guided by current recommendations and based on a multidisciplinary clinical evaluation of the individual patient. However, no meaningful comparison of survival according to treatment regimen could be performed due to the variations in and many combinations of the non‐surgical treatments administered over the course of disease in this study population.

The median follow‐up time was 82 (IQR, 20–117) months. Overall, 111 patients (111/190 = 58.4%) were still alive, and 88 of those patients (88/111 = 79.3%) exhibited no evidence of disease at the last follow‐up. Among the 77 (77/190 = 40.6%) patients who died during follow‐up, advanced GEP‐NEN malignant disease was determined as the cause of death in 57 (57/77 = 74.0%) patients. The remaining 20 patients (26.0%) died from various unrelated causes.

**FIGURE 3:**
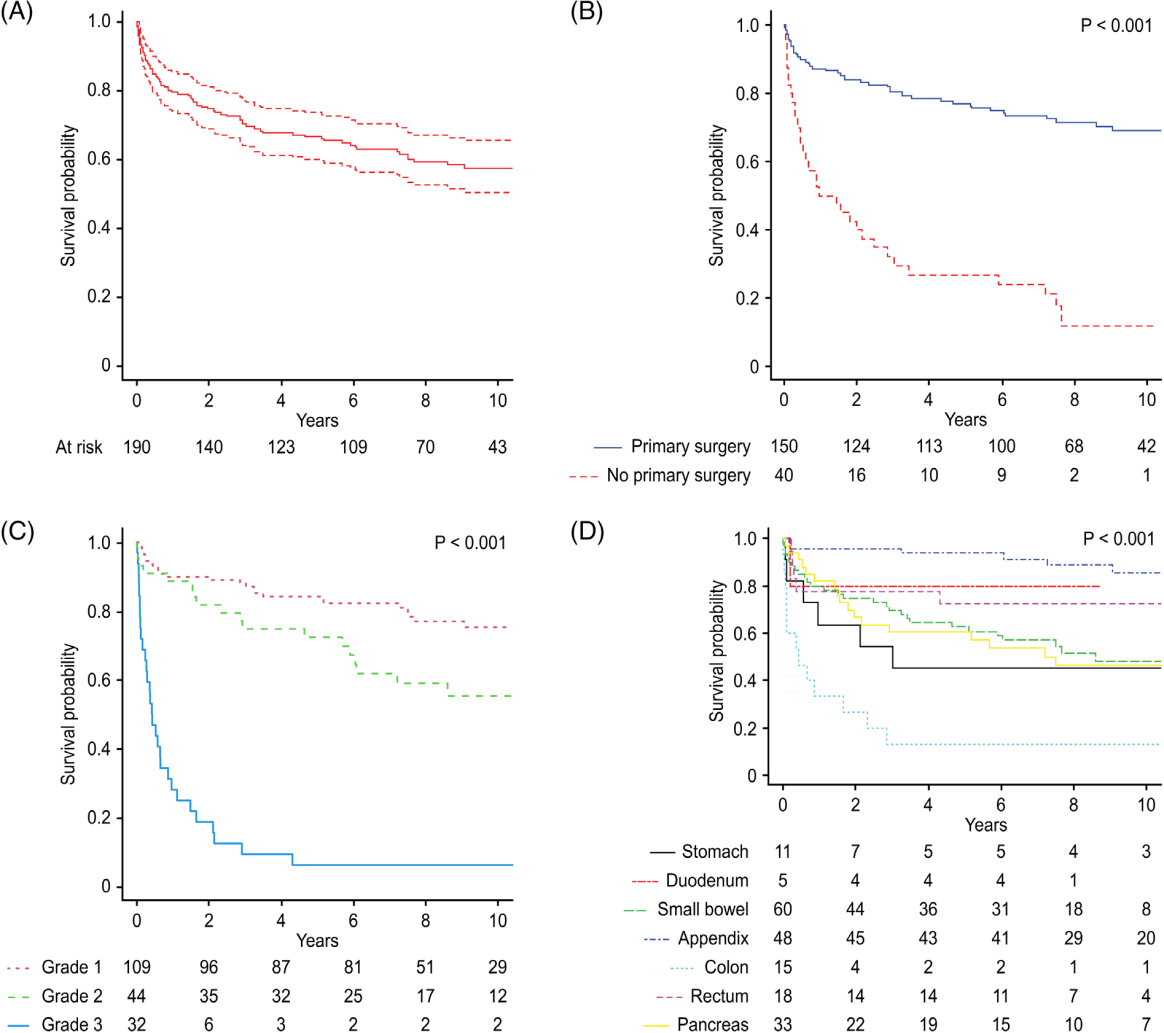
Overall survival (OS) with 95% CI (panel A) grouped according to whether primary surgery was performed or not (panel B) base on WHO tumor grades 1–3 (panel C) and on localization of the primary neuroendocrine tumor (panel D)

The median OS time was 183 (95% CI 122–243) months with 5‐ and 10‐year OS rates of 66% and 57%, respectively (Figure 3(A)). Survival was significantly better in patients who underwent primary surgery at the time of diagnosis (*p* < .001) (Figure 3(B)). Moreover, tumor grade was significantly associated with OS, and a poor prognosis was seen in those with WHO grade 3 tumors (*p* < .001) (Figure 3(C)). In the univariable survival analysis, primary tumor location was statistically significant (*p* < .001), as better survival was observed in patients with tumors in the appendix, duodenum, or rectum, while worse survival was observed in patients with primary colon tumors (Figure 3(D)). As shown in Table 2, survival was similar in both sexes (*p* = .381). However, younger patients (i.e., median age ≤ 60 years) had significantly better OS (*p* < .001) than those above 60 years of age, and patients with incidentally discovered tumors had better survival (*p* = .003) than symptomatic patients. In addition, survival differed significantly (*p* < .001) among UICC stages, and a poor prognosis was observed in stage IV patients.

In the Cox multivariable survival analysis, only age, WHO tumor grade, and primary surgical treatment were independent predictors of survival (Table 3). Regarding tumor grade as a prognostic factor, a significant difference was evident between those with malignant grade 3 tumors and those with differentiated grade 1 and 2 tumors (Table 3).

**TABLE 3:** (A) Factors of importance for overall survival (OS) (B) disease‐specific survival (DSS)

(A)				
Variable	Univariable analysis		Multivariable analysis	
	*p*‐value	Hazard ratio (95%CI)	*p*‐value	Hazard ratio (95%CI)
Gender	.538	1.15 (0.73–1.82)		
Age ≤ 60/>60	**<.001**	5.91 (3.39–10.29)		
Age, continuous	**<.001**	1.06 (1.05–1.08)	**<.001**	1.06 (1.0–1.08)
Symptomatic	**.001**	2.62 (1.51–4.55)		
Primary tumor location	**<.001**			
Stomach	Ref			
Duodenum	.295	0.32 (0.04–2.69)		
Small bowel	.647	0.81 (0.34–1.97)		
Appendix	**.002**	0.18 (0.06–0.53)		
Colon	**.021**	3.15 (1.19–8.37)		
Rectum	.154	0.43 (0.13–1.38)		
Pancreas	.874	0.93 (0.37–2.34)		
WHO grade 1–3	**<.001**		**<.001**	
Grade 1	Ref		Ref	
Grade 2	**.009**	2.19 (1.22–3.94.)	.111	1.62 (0.90–2.91)
Grade 3	**<.001**	12.2 (7.0–21.5)	**<.001**	8.32 (4.56–15.21)
Primary surgical treatment	**<.001**	0.20 (0.12–0.31)	**<.001**	0.43 (0.26–0.73)
UICC stage I–IV	**<.001**			
Stage I	Ref			
Stage II	**.003**	3.92 (1.58–9.74)		
Stage III	**.001**	3.60 (1.67–7.77)		
Stage IV	**<.001**	8.71 (4.45–17.0)		

(B)				
Variable	Univariable analysis		Multivariable analysis	
	*p*‐value	Hazard ratio (95%CI)	*p*‐value	Hazard ratio (95%CI)
Gender	.769	0.93 (0.55–1.56)		
Age ≤ 60/>60	**<.001**	4.36 (2.38–7.99)		
Age, continuous	**<.001**	1.05 (1.03–1.07)	**<.001**	1.04(1.02–1.06)
Symptomatic	**<.001**	8.36 (3.02–23.11)		
Primary tumor location	**<.001**			
Stomach	Ref			
Duodenum	.967	1 (0–155)		
Small bowel	.350	0.63 (0.23–1.67)		
Appendix	**.002**	0.033 (0.004–0.28)		
Colon	**.030**	3.19 (1.12–9.11)		
Rectum	.179	0.41 (0.11–1.51)		
Pancreas	.920	0.95 (0.35–2.59)		
WHO grade 1–3	**<.001**		**<.001**	
Grade 1	Ref		Ref	
Grade 2	**<.001**	4.82 (2.13–10.91)	**.002**	3.67 (1.62–8.33)
Grade 3	**<.001**	32.1 (14.7–70.0)	**<.001**	22.2 (9.8–50.4)
Primary surgical treatment	**<.001**	0.14 (0.08–0.24)	**<.001**	0.32 (0.18–0.58)
UICC stage I–IV	**<.001**			
Stage I	Ref			
Stage II	**.022**	8.08 (1.35–48.4)		
Stage III	**.002**	10.9 (2.35–50.3)		
Stage IV	**<.001**	44.7 (10.8–185)		

*Note*: Bold letters in the Table to emphasise *p*‐values that are statistically significant (e.g. *p* < 0.001 vs *p* = 0.47).

DSS rates of 73.1% and 68.1% were observed at 5 and 10 years, respectively. A median DSS was not calculated for the whole cohort. During the follow‐up, 57 patients (30.0%) died from proven advanced GEP‐NEN disease, and the best DSS was observed in patients with appendiceal and duodenal NENs; this was in contrast to a relatively dismal prognosis in patients with colon‐NENs, as indicated by the OS in Figure 3(D). Intermediate survival was achieved in patients whose primary tumors were located in the rectum, small intestine, stomach, and pancreas, and a better DSS was observed in patients with tumors of rectal and small intestinal origins. Notably, even patients with pancreatic‐NENs had at least a 50% median survival at 10 years.

**FIGURE 4:**
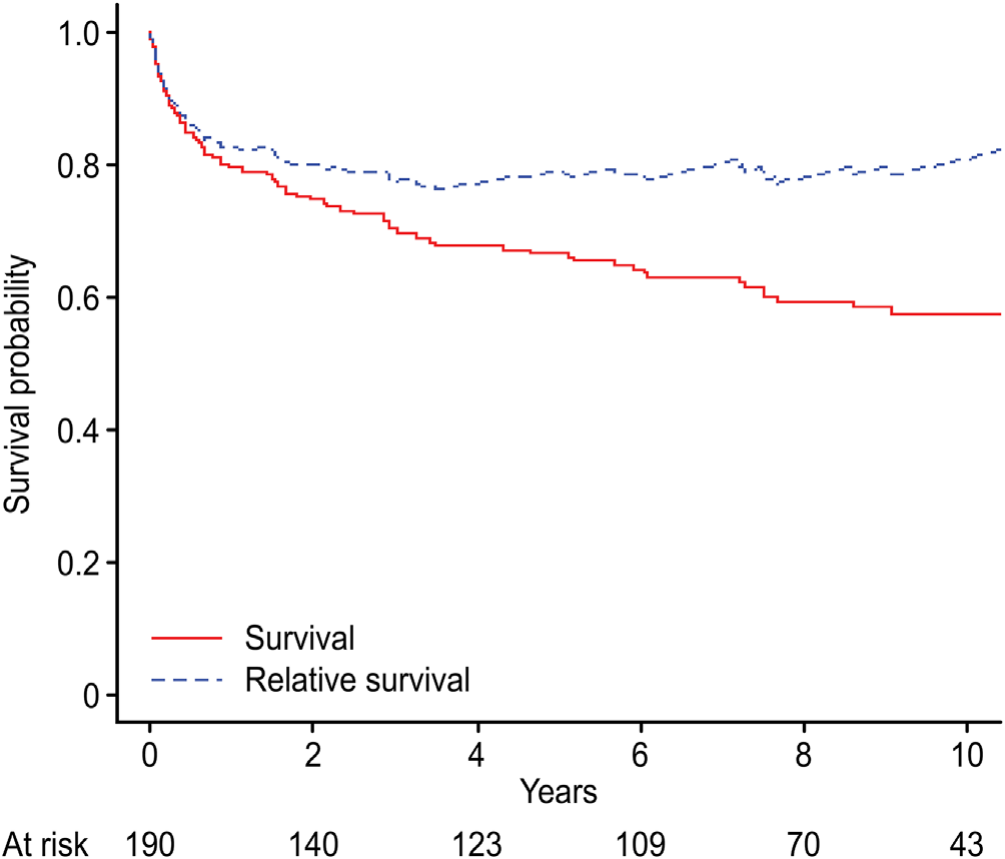
Overall survival (OS) and relative survival of the patient cohort. Notably, the two curves have a similar pattern, particularly during the early time period of 2–3 years

In Figure 4, the estimated OS (Kaplan‐Meier plot) is depicted alongside the calculated relative survival curve for age and sex‐adjusted populations without a diagnosis of GEP‐NENs. The curves are relatively comparable during the first 2 years. After that, the relative survival curve plateaued, which shows that the excess deaths attributed to the disease mainly occurred during the first 2 years.

## 4 DISCUSSION

GEP‐NENs comprise a heterogeneous group of tumors.^1^, ^3^ Many factors including age, symptomatic disease, primary tumor location, disease stage, tumor grade, and primary surgical treatment are relevant for prognostic prediction.^6^, ^8^, ^10^, ^28^, ^29^

In this study, the most common tumor origins were the small intestine (31.6%), appendix (25.3%), and pancreas (17.4%). This is in agreement with a Canadian population‐based study, which indicated the small bowel as the predominant location, although pancreas NENs were not included in that report.^30^ In a recent national survey from Iceland that studied a national population comparable to our hospital's regional population (i.e., ≈380 000), the tumor origin distribution was slightly different, with 23.1% occurring in the small bowel, 30.8% in the appendix, and 9.6% in the pancreas.^6^ These authors reported an incidence of 3.85/100 000 from 2000 to 2014, which is lower than the incidence of 5.83/100 000 reported in our previous study^4^ and by others^5^ during a similar time period.

The relative indolent nature of this group of tumors is reflected in a promising long‐term prognosis, with 5‐ and 10‐year DSS rates of 73.1% and 68.1%, respectively, and 5‐ and 10‐year OS rates of 66% and 57%, respectively. However, as shown in this study, survival is related to several factors many of which cannot be moderated. Moreover, the increasing gap (see Figure 4) between the OS curve and the calculated relative survival curve, particularly during the first 2 years, may indicate that an excess mortality risk in GEP‐NEN patients compared with the age‐ and gender‐adjusted general population, is mainly attributed to the early time period after a GEP‐NEN diagnosis is confirmed.

Decreased survival has been associated with older age, the presence of symptoms, primary tumor location, tumor grade, and disease stage.^6^, ^8^, ^10^ This study confirms the prognostic value of age for both OS and DSS, with a 4‐ to 5‐fold increased risk of death in patients older than 60 years (i.e., median age). While survival also varied according to primary tumor location, only the appendix (with an excellent prognosis) and colon (with a more dismal prognosis) sites were significantly associated with prognosis. However, the primary tumor location did not retain its independent association with prognosis in the multivariable analysis, which was also the case for the disease stage. This is partly in contrast to recent population‐based studies,^5^, ^8^ which reported that sex, tumor differentiation, stage, and primary site were independent predictors of OS. However, attention should be paid to the differences among these studies, as they included patient populations with tumor locations outside the gastrointestinal‐pancreatic sites, contained reporting bias due to a national registry, and considered different definitions to describe tumor differentiation. Thus, caution is warranted when making direct comparisons.

The prognostic relevance of tumor grading, as first proposed in Europe more than a decade ago^31^ and eventually embraced by clinicians worldwide,^32^, ^33^, ^34^ is also supported by observations in this study. As shown in our research, the distribution of grades varied according to primary tumor site (Figure 1) and disease stage (Figure 2(A)), which is in accordance with observations reported by Fitzgerald et al.^35^ based on the evaluation of 39 454 GEP‐NEN patients from the National Cancer Data Base (NCDB) in the US. However, the NCDB does not report Ki67 staining‐based grades, which is important to reliably define tumor grade, as noted by the authors.^35^ Several limitations, including inconsistent criteria for tumor grading and tumor differentiation, and the fact that only malignant tumors are reported in the registry, hinder the utility of the results provided in national databases and registries.^6^, ^8^, ^35^

As seen in our study, NENs in the stomach, colon, and pancreas were more often grade 2/3 tumors, and grade 2/3 tumors were encountered more frequently in stage III/IV patients compared with stage I/II patients. In the multivariable analysis, tumor grade was an independent predictor of prognosis, unlike both primary tumor site and disease stage. Recently, the WHO tumor grade (1–3) has gained additional attention and is currently a standard part of the primary evaluation of GEP‐NEN patients.^20^ Moreover, the finding of a higher grade (i.e., WHO grade 3) in metachronous liver metastases in GEP‐NEN patients compared with a low‐grade primary tumor will further add prognostic value by indicating a poor prognosis.^36^, ^37^

Tumor grade not only serves as a prognostic factor but may also serve as a predictive factor for the selection of GEP‐NEN patients for chemotherapy treatment,^34^ particularly in cases with a well‐differentiated morphology despite a high Ki67 index (>55%).^38^

In line with long‐standing treatment principles, surgical treatment was used whenever feasible.^10^, ^21^, ^22^, ^39^ The high proportion of patients (78.9%) who were surgically treated, including 66.3% treated with curative intent in the total cohort, is partially explained by the assumption that removal of the primary tumor even in stage IV patients may be beneficial.^40^ The decision to operate on an individual patient is based on many aspects, including patient‐ and disease‐related factors. In recent years, patients undergoing curative or debulking surgery have undergone preoperative ^68^Ga DOTATOC‐PET/CT or ^18^FDG‐PET/CT according to tumor grade. Studies have demonstrated the superior sensitivity of SRI‐PET in the detection of metastasis and primary tumors and in the evaluation of disease burden, and this methodology can have a significant impact on patient management.^41^, ^42^, ^43^, ^44^ This may improve the OS of patients undergoing surgery due to more accurate preoperative staging.

Selection bias is likely to occur when surgically treated and non‐surgically treated patients are compared. Moreover, arguments for a liberal approach to surgery are mostly based on retrospective observational studies, and no randomized prospective study has shed light on this topic thus far. Several recent studies have promoted removal of the primary tumor and claim that this strategy is beneficial to most patients with GEP‐NENs.^40^, ^45^, ^46^, ^47^ An aggressive approach is suggested for patients with several liver metastases to achieve >70% cytoreduction, which may translate into long‐term survival.^48^ In contrast, a watch‐and‐wait approach is considered for other patients, including stage IV patients with small intestinal NENs^49^ and those with WHO grade 1 non‐functional pancreatic‐NETs smaller than 2 cm.^50^ In addition, the recommended non‐surgical approach may be replaced by proton pump inhibitor (PPI) treatment in most MEN‐1 patients with a gastrinoma smaller than 2 cm.^51^

One strength of this study is that it provides observations from a population‐based patient cohort, with patients diagnosed and treated consecutively and consistently according to previously described guidelines and criteria.^4^ Both the clinical and morphological diagnoses and workup were consistently completed during the study period according to recommended national and international guidelines and criteria and were managed using a multidisciplinary team approach. A relatively long follow‐up time with a median of almost 7 years, complete follow‐up regarding outcomes, and no missing prognostic factor data included in the evaluation added to the strength of this observational study.

Some limitations of this study, including its retrospective nature, should be mentioned. Due to the relatively low incidence of GEP‐NENs, the number of patients was limited. As already noted, due to the heterogeneity of these patients and the variations in treatment regimens, evaluations are challenging. However, this may partly be remedied in that during the last decade, the universal criteria for UICC staging^12^, ^13^ and tumor grading^13^, ^52^ have been implemented in routine practice, which allows for more feasible and useful comparisons between various studies. This study population mirrors largely a population‐based unselected population, with a very low number of patients (*n* = 14) excluded from the final evaluation due to criteria explained previously. Likely, this selection bias would hardly impact any main conclusions drawn from this study.

Furthermore, we fully recognize the inherent risk of misinterpretations by comparing small subgroups based on underpowered calculations.

This study confirms the heterogeneity of patients with GEP‐NENs in both localization and stage but also in the aggressiveness of the disease. Although many factors are associated with prognosis, only age, tumor grade, and primary surgical treatment showed independent prognostic importance for OS and DSS. However, these factors and endpoints are rather crude measures. To further tailor treatment approaches to individual patients in terms of stage and type of disease, novel insights beyond established clinical patterns and morphological criteria are warranted. In this regard, recent observations that even pathologically homogeneous tumors, such as small intestinal NENs, can be further subdivided into two different subtypes of tumors are interesting.^53^ Moreover, the application of observed epigenetic modifications to serve as potential prognostic biomarkers, or even as therapeutic targets, may present novel opportunities in the future.^54^ In addition, metabolic grading using FDG‐PET^55^ and the development of artificial intelligence (AI) imaging approaches (i.e., tumor heterogeneity) seem to be promising prognostic stratification tools.^56^ Finally, knowledge‐based supportive care for GEP‐NEN patients, including those with a lengthy course trajectory, and even those with advanced disease, should not be overlooked.^57^

## CONFLICT OF INTEREST

The authors have stated explicitly that there are no conflicts of interest in connection with this article. All authors have reviewed and consented to the submitted final version of this manuscript.

## AUTHORS' CONTRIBUTIONS

All authors had full access to the data in the study and take responsibility for the integrity of the data and the accuracy of the data analysis. *Conceptualization*, J.A.S.; *Data Curation*, J.A.S., J.T.K., D.L., O.M.S., M.A.‐S., T.M.H., H.G., L.N.K., E.G., K.S.; *Methodology*, J.A.S., J.T.K., D.L., L.N.K., E.G., K.S.; *Investigation*, J.A.S., M.A.‐S., T.M.H., K.S.; *Formal Analysis*, J.A.S., J.T.K., D.L., K.S.; *Resources*, J.A.S., O.M.S., M.A.‐S., T.M.H., H.G., L.N.K., E.G., K.S.; *Writing ‐ Original Draft*, J.A.S., D.L., O.M.S., H.G., L.N.K., E.G., K.S.; *Writing ‐ Review & Editing*, J.A.S., J.T.K., D.L., O.M.S., M.A.‐S., T.M.H., H.G., L.N.K., E.G., K.S.; *Visualization*, J.A.S., M.A.‐S.; *Supervision*, J.A.S.; *Funding Acquisition*, J.A.S.; *Project Administration*, J.A.S, O.M.S.; *Software*, J.A.S., J.T.K.; *Validation*, J.A.S., D.L., M.A.‐S., T.M.H., H.G., L.N.K., E.G., K.S.

## ETHICAL STATEMENTS

This study was approved by the Institutional Review Board (Study # 2011/1659‐16) according to the general guidelines provided by the Regional Committees for Medical and Health Research Ethics. This approval includes a consent to publish the results .

## Data Availability

The data that support the findings of this study are available from the corresponding author upon reasonable request.
